# Level of discharge readiness and influencing factors in ischaemic stroke patients: a descriptive, cross-sectional study

**DOI:** 10.3389/fneur.2025.1683780

**Published:** 2025-10-31

**Authors:** Xiaolu Bai, Lei Gao, Hongli Li, Ruiling Li, Ying Zhang, Lingyu Han, Dandan Liang, Yining Wang, Yurui Zhang, Wenjia Yang

**Affiliations:** ^1^Huaihe Hospital of Henan University, Kaifeng, Henan, China; ^2^College of Medicine, Technology & Media University of Henan Kaifeng, Kaifeng, Henan, China; ^3^The First Affiliated Hospital of Henan University, Kaifeng, Henan, China; ^4^Guangzhou Nanyang Polytechnic College, Guangzhou, Guangdong, China; ^5^Medical College of Jinzhou Medical University, Jinzhou, Liaoning, China

**Keywords:** patients with ischaemic stroke, discharge readiness, influencing factors, level of discharge readiness, transitional care

## Abstract

**Objectives:**

This study aims to evaluate readiness for hospital discharge (RHD) levels in ischemic stroke patients and identify influencing factors, providing evidence for targeted post-discharge interventions to improve secondary prevention outcomes.

**Materials and methods:**

In this context, we conducted a descriptive cross-sectional study to investigate and analyse the factors influencing discharge readiness among 220 patients with ischaemic stroke from 1 June to 31 December 2024 in a tertiary hospital in China. Statistical analyses were performed using SPSS 26.0, with significant results visualised in GraphPad Prism 9.0. Descriptive statistical analysis of frequency, component ratio, and mean±standard deviation was conducted using a self-made general sociological data and disease characteristics questionnaire, the Readiness for Hospital Discharge Scale (RHDS), and the Quality of Discharge Teaching Scale (QDTS), and single-factor analysis was performed by independent sample t-test and one-way ANOVA. Pearson correlation analysis was used to describe the relationship between the two scales. Single-factor and correlation analyses of statistically significant variables were included in the equation, and multiple stepwise linear regression analysis was performed to test, interpret, and analyse the collected data.

**Results:**

The readiness score of ischaemic stroke patients, as measured by the readiness scale, was 6.13 ± 0.75 points. Results from multiple linear regression analysis indicated that the quality of discharge guidance, length of hospital stay, presence of comorbid conditions, frequency of stroke occurrence, and mRS scores were independent factors influencing caregiver readiness (*p* < 0.05).

**Conclusion:**

Readiness for discharge among ischaemic stroke patients is inadequate and positively correlated with the quality of discharge guidance. Patients with shorter hospital stays, a higher number of comorbid conditions, increased frequency of stroke occurrence, and higher mRS scores exhibited lower readiness for discharge. These findings suggest that healthcare professionals should enhance the quality of discharge education and provide targeted interventions for patients with shorter hospitalisation and more severe conditions. Additionally, establishing a secondary prevention support mechanism is essential to improve discharge readiness and ensure better post-discharge outcomes for ischaemic stroke patients. This will ensure their safe transition from hospital to home.

## 1 Introduction

Stroke is the second leading cause of global mortality and third leading cause of disability-adjusted life years worldwide (1–3). The dual burden of lethality and chronic impairment solidifies stroke as a critical public health priority, particularly given its rising incidence among younger populations (4, 5). Among stroke subtypes, ischaemic stroke accounts for 85% of all stroke types and is characterised by high morbidity, mortality, disability and recurrence (6, 7). Although advancements in thrombolytic therapies and endovascular interventions have significantly reduced acute-phase mortality, neurological deficits, including impaired language function, dysphagia, cognitive decline, and motor disabilities, frequently persist beyond the acute stabilisation period (8). Among those who survive ischaemic stroke, the disability rate is as high as 40% (9). They are also unable to work to varying degrees and need others to take care of them in their daily lives, placing a heavy burden on families and society (10, 11).

However, most patients with ischaemic stroke are discharged after a short period of acute hospitalisation. When patients are discharged from the hospital, there are often problems, such as incomplete recovery of physical function, insufficient knowledge and skills of continuing nursing, and insufficient cognitive and psychological preparation. The nursing needs and rehabilitation burden left by patients after discharge also extend directly from the hospital to home.

Studies have shown that the transition from hospital to home is a critical period for patient rehabilitation and adaptation (12). During this period, patient discharge readiness is very important, and the quality of discharge readiness often affects transition outcomes after discharge (13). Discharge readiness refers to the readiness of medical institutions to assess a patient’s recovery ability and perceived discharge before discharge, as well as the safety of the transition period after discharge, and is often used as an outcome indicator to identify patients’ unmet discharge needs (14). If a patient does not have sufficient self-care and knowledge at the time of discharge, this will affect the later recovery of patients, increase the incidence of complications, and increase the re-entry rate of unplanned admissions (15).

Therefore, this study investigated the discharge preparation period of ischaemic stroke patients and explored its influencing factors to provide a basis and suggestions for the formulation of targeted intervention measures in clinical practise to ensure the safe transition of ischaemic stroke patients from discharge to family.

## 2 Methods

### 2.1 Study design and setting

This cross-sectional study was conducted at Huaihe Hospital of Henan University, Kaifeng, Henan Province, China. Participant recruitment occurred1 June to 31 December 2024. The research team comprised three neurologists and six neurology-specialised nurses. Prior to administering the survey to patients with ischaemic stroke, all members participated in standardised training to ensure data quality and consistency.

### 2.2 Transition theory

Guided by Meleis’ Transition Theory (16), which conceptualises transition as a process of psychological and social adaptation during changes in health or illness status, this study emphasises the importance of preparedness in physical, psychological, and social support domains for individuals moving from hospital to family or community settings. The present research examines the readiness for hospital discharge among ischaemic stroke patients and its influencing factors, with the aim of informing the development of clinical interventions to ensure safety during the transition period after discharge.

### 2.3 Participants

In this study, the inclusion and exclusion criteria were as follows: Criteria for inclusion of participants:

1. ≥ 18 years old.
2. The current clinical diagnosis is ischaemic stroke, requiring acute care.
3. Can independently sign an informed consent form.
4. Basic listening, speaking, reading, and writing functions.

Criteria for excluding participants:

1. Patients in critical condition requiring rescue.
2. Patients with severe mental and psychological conditions who cannot cooperate.

Withdrawal criteria:

If patients experienced any discomfort in the process of filling in the questionnaire, they could unconditionally choose to quit. Patient refusal would not have any adverse effects on others.

### 2.4 Study sample

According to the calculation of Gpower sample size, the type 1 error (*α*) value was set to 0.05; the power (1−*β*), 0.8; and the median value of influence factor *δ*, 0.15. Gpower calculated that the sample size was about 210 cases, and considering the attrition rate of 5%–10%, the final sample size would be 220 cases.

### 2.5 Measurements

#### 2.5.1 General sociological and disease characteristics

The author developed the general sociological and disease characteristics questionnaire by referring to the literature and combining the disease characteristics of clinical patients. The questionnaire included gender, age, education, occupation, per capita monthly household income, insurance and marital status, length of stay, number of ischaemic strokes, combined with other disease types, hemiplegia, legacy dysfunction, indwelling gastric tube, stroke frequency, family history, type of obstacle, and modified Rankin Scale (mRS) scores. The specific survey questionnaire items are described in the Supplementary material.

#### 2.5.2 Readiness for hospital discharge scale

This scale was compiled by Marianne et al. (17). The Chinese RHDS introduced, translated, and revised by Lin et al. in 2014 was selected (18). The Chinese RHDS has 12 items and 3 dimensions. The personal status dimension includes 1–3 questions, the adaptability dimension includes 4–8 questions, and the anticipatory support dimension includes 9–12 questions. Scores for individual items range from 0 to 10, and the total score for the 12-item readiness for hospital discharge scale ranges from 0 to 120. The higher the score, the better the patient’s readiness for discharge. A score of 0 to 7 for all items is defined as inadequate readiness for discharge, 7 to 8 as moderate readiness, 8 to 9 as high readiness, and > 9 as high readiness (19). The Cronbach’s alpha of the RHDS is 0.89, and the content validity index is 0.88 (17). The specific survey questionnaire items are described in the Supplementary material.

#### 2.5.3 Quality of discharge teaching scale

The QDTS, developed by Weiss (20), consists of three dimensions, including content needed, content obtained, guiding skills, and effects. There are six groups of items matched by the dimensions of the required content and the obtained content, and the difference between the score of the obtained content and the score of the corresponding required content can be used to judge whether the discharge guidance meets the needs of patients. The scale is scored from 0 to 10. The quality of discharge guidance for patients is judged by the total score of the content dimension, guidance skills, and effect dimension. The higher the score, the better is the quality of guidance. Scores of 0–7 are defined as insufficient quality of discharge guidance, 7–8 as medium quality of discharge guidance, 8–9 as high quality of discharge guidance, and > 9 as high quality of discharge guidance (20). A multicentre cross-sectional study on the discharge guidance quality of 602 hospitalised patients showed that the Cronbach’s *α* coefficient of the questionnaire was 0.92, and the subscales ranged from 0.88–0.94 (21). The specific survey questionnaire items are described in the Supplementary material.

### 2.6 Analytical strategy

The researchers accessed the retrospective survey datasets for analysis on 15 January 2025, with verification procedures completed by 30 January 2025. Original data collection had occurred from 1 June to 31 December 2024. The researchers only had access to fully anonymized datasets throughout the study period. No personally identifiable information was available during or after data collection. After the electronic questionnaire was numbered in SPSS 26.0. Frequency, component ratio, and mean ± standard deviation were used for descriptive statistical analysis. Independent sample t-test and one-way ANOVA were used to analyse the relationship between nominal data and hospital readiness scores. Pearson correlation analysis was used to analyse the relationship between interval data and discharge readiness scores, and was used to describe the relationship between the two scales. Univariate and correlation analyses of statistically significant variables were included in the equation for multiple stepwise linear regression analysis. Statistically significant results were plotted in GraphPad Prism 9.0.

### 2.7 Ethical considerations

All respondents were informed of the aims and objectives of the study and were provided the written consent form in the questionnaire itself. Participants were aware that their participation was voluntary. The confidentiality of the participants was ensured. This study was approved by the Ethics Committee of Huaihe Hospital, Henan University (Approval number: 2023(207)).

## 3 Results

### 3.1 The level of discharge readiness of patients with ischaemic stroke

The overall score of discharge readiness of patients with ischaemic stroke was 73.56 ± 9.03, with an individual entry score of 6.13 ± 0.75, which was composed of three dimensions: (1) Personal status: physiologic indicators (pain, physical strength, energy, etc.) to evaluate patients at discharge. (2) Anticipatory support: Evaluate the social and family support and help that patients can obtain in medical care and daily life after returning to the family. (3) Adaptive ability: The ability to perform daily care and functional exercises was evaluated at discharge and after returning home. The scores for personal status, anticipatory support, and adaptability were 7.48 ± 1.12, 6.07 ± 0.82, and 5.37 ± 0.83 points, respectively. Detailed information is provided in Table 1.

**Table 1:** Scores of various dimensions of discharge readiness of patients with ischaemic stroke (*n* = 220).

Variable	Number of entries	Total score	Single entry score	Individual item sorting
		Mean ± SD	Mean ± SD	
Discharge readiness	12	73.56 ± 9.03	6.13 ± 0.75	
Personal status	3	22.44 ± 3.37	7.48 ± 1.12	1
Anticipatory support	4	24.28 ± 3.28	6.07 ± 0.82	2
Adaptability	5	26.84 ± 4.17	5.37 ± 0.83	3

### 3.2 The general sociological characteristics of patients with ischaemic stroke and their readiness for discharge

Univariate analysis was conducted using general sociological characteristics as the independent variable and the total score of discharge readiness of patients with ischaemic stroke as the dependent variable. The results showed that the scores of patients of different ages and incomes (yuan/month) differed significantly (*p* < 0.05). Details are presented in Table 2.

**Table 2:** Comparison of discharge readiness differences among ischaemic stroke patients with different sociological characteristics (*n* = 220).

Variable	Participants	RHDS	*t*/F	*p*
	*n* (%)	Mean ± SD		
Gender			−1.437^1)^	0.152
Male	110 (50.00)	72.69 ± 8.43		
Female	110 (50.00)	74.44 ± 9.56		
Age (years old)			2.752^1)^	0.006
≤60	124 (56.36)	75.02 ± 8.42		
>60	96 (43.64)	71.69 ± 9.47		
BMI (kg/m^2^)			1.844^2)^	0.140
≤18.4	8 (3.64)	71.13 ± 9.63		
18.5–23.9	58 (26.36)	75.76 ± 8.56		
24–27.9	123 (55.91)	73.13 ± 9.10		
≥28.0	31 (14.09)	71.81 ± 9.09		
Education level			0.801^2)^	0.450
Junior high school and below	107 (48.63)	73.64 ± 8.75		
High school or technical secondary school	83 (37.73)	72.84 ± 9.27		
College and above	30 (13.64)	75.27 ± 9.38		
Occupation			1.047^2)^	0.353
Work	37 (16.82)	75.24 ± 6.60		
Retire	64 (29.09)	72.55 ± 9.41		
No job or laid off	119 (54.09)	73.59 ± 9.45		
Income (yuan/month)			11.517^2)^	<0.000
<3,000	67 (30.45)	71.37 ± 8.88		
3,000–5,000	97 (44.10)	72.35 ± 9.97		
>5,000	56 (22.45)	78.29 ± 5.03		
Marital status			0.515^2)^	0.673
Unmarried	7 (3.18)	74.00 ± 10.46		
Married	166 (75.46)	73.92 ± 8.92		
Divorced	13 (5.91)	71.15 ± 6.38		
Widowed	34 (15.45)	72.65 ± 10.24		
Medical insurance			0.658^2)^	0.519
Resident medical insurance	99 (45.00)	72.77 ± 8.86		
Employee medical insurance	96 (43.64)	74.21 ± 9.24		
Other medical insurance	25 (11.36)	74.04 ± 8.94		

1) *t* value; 2) *F* value; SD, standard deviation.

**Figure 1:**
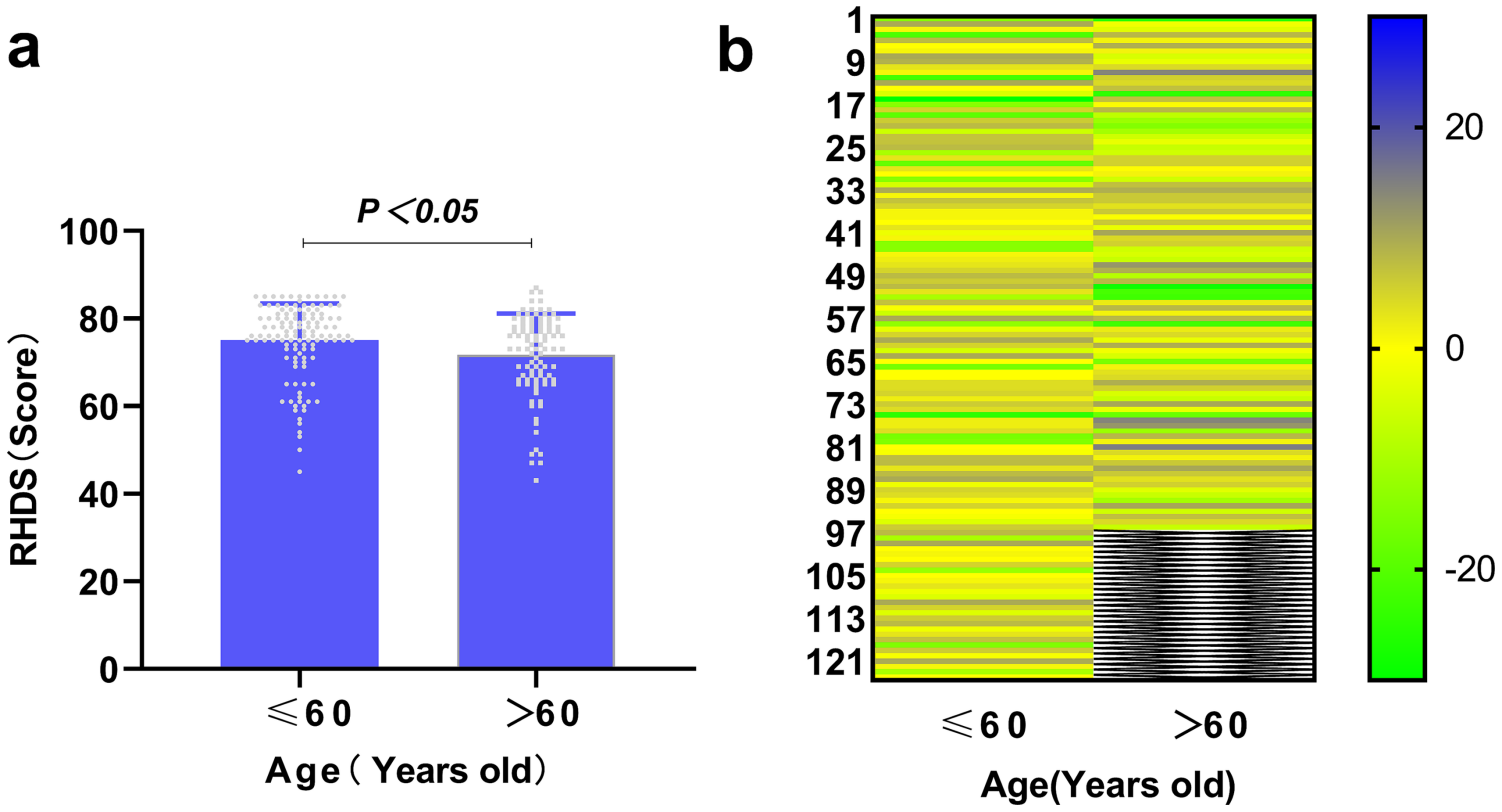
Comparison of discharge readiness in ischaemic stroke patients by age. **(a)** The X-axis represents age subgroups, while the Y-axis indicates readiness for hospital discharge scores (mean ± SD). A significant intergroup difference was observed (independent samples *t*-test, *p* < 0.05). **(b)** Heatmap visualising the distribution of individual RHDS scores within each independent age group. Each row represents one unique individual from the ≤60 age group and one unique individual from the >60 age group. The two groups are displayed side-by-side for visual comparison of score distribution patterns. Scores were converted to Z-scores normalised within each group. The colour gradient represents Z-score values (blue: highest scores; yellow: scores near the group mean; green: lowest scores).

**Figure 2:**
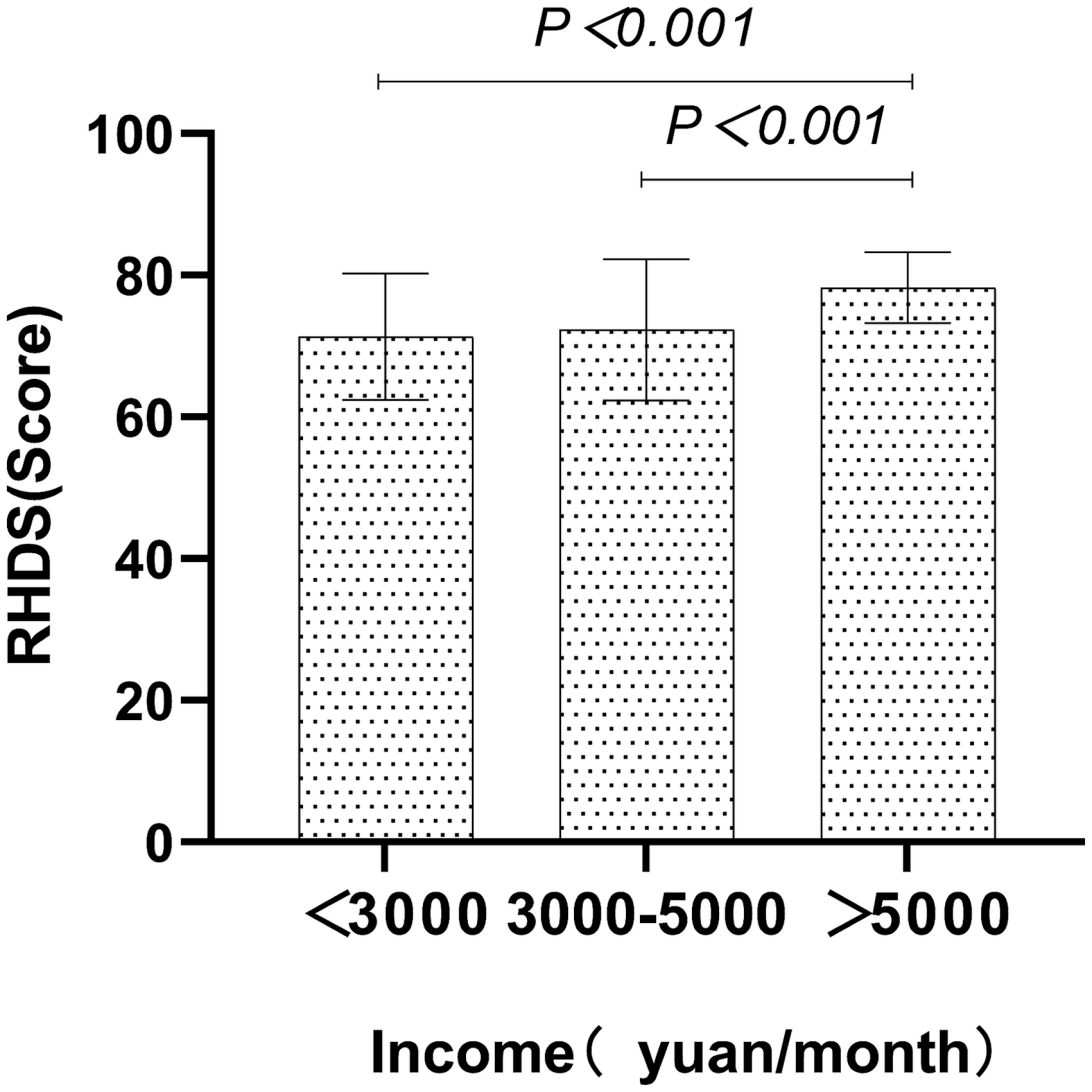
Comparison of discharge readiness in ischaemic stroke patients by income. The X-axis represents the subgroups of income, and the Y-axis represents readiness for hospital discharge scores (mean ± SD). Significant intergroup differences were observed (one-way ANOVA, *p* < 0.001).

Figures 1, 2 show the sociological characteristics with statistically significant differences.

### 3.3 The disease characteristics of patients with ischaemic stroke and their readiness for discharge

**Figure 3:**
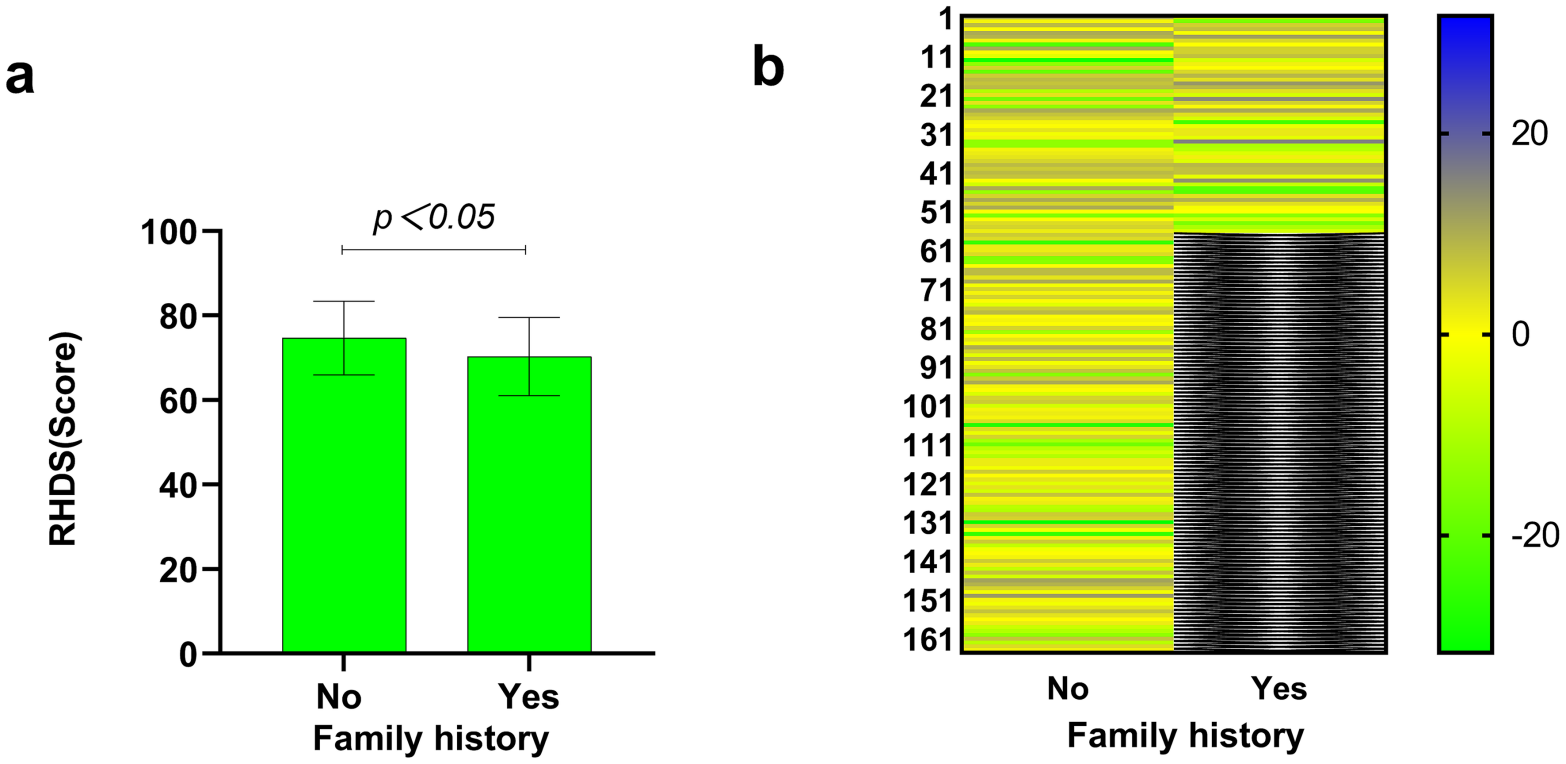
Comparison of discharge readiness in ischaemic stroke patients by family history status. **(a)** The X-axis shows subgroups based on family history, and the Y-axis displays readiness for hospital discharge scores (mean ± SD). A significant intergroup difference was observed (independent samples *t*-test, *p* < 0.05). **(b)** Heatmap visualising the distribution of individual RHDS scores within each independent family history group. Each row represents one unique individual from the no family history group and one unique individual from the family history group. The two groups are displayed side-by-side for visual comparison of score distribution patterns. Scores were converted to Z-scores normalised within each group. The colour gradient represents Z-score values (blue: highest scores; yellow: scores near the group mean; green: lowest scores).

**Figure 4:**
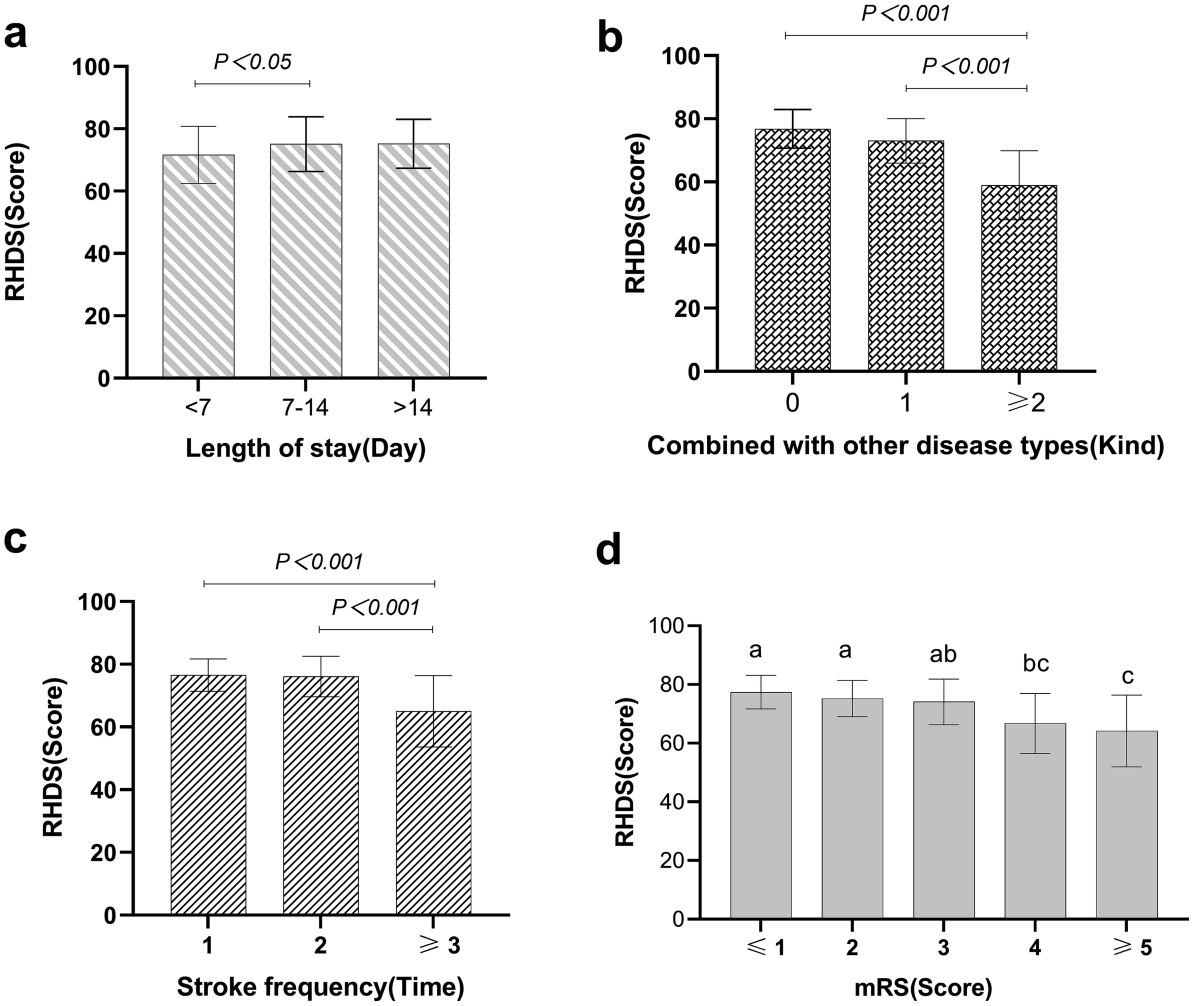
Comparison of discharge readiness in ischaemic stroke patients with statistically significant disease characteristics. In panels **(a–d)**, the X-axis represents the subgroups of disease characteristics demonstrating statistical significance, while the Y-axis represents readiness for hospital discharge scores (mean ± SD). Significant intergroup differences were observed (one-way ANOVA, *p* < 0.05). In panel **(d)**, means labelled with identical letters showed no significant difference (*p >* 0.05), whereas those with distinct letters exhibited statistically significant differences (*p* < 0.05).

Disease characteristics were taken as independent variables, and the total score of discharge readiness of patients with ischaemic stroke was taken as the dependent variable. Univariate analysis was conducted, and the results showed significant differences (*p* < 0.05) in mRS scores, length of stay, comorbidity type, stroke frequency, and family history. The details are shown in Table 3. Figures 3, 4 show the disease characteristics with statistically significant differences.

**Table 3:** Comparison of discharge readiness differences among patients with ischaemic stroke with different disease characteristics (*n* = 220).

Variable	Participants	RHDS	*t/F*	*p*
	*n* (%)	Mean ± SD		
Length of stay			4.235^2)^	0.016
<7 days	97 (44.09)	71.60 ± 9.18		
7–14 days	107 (48.64)	75.10 ± 8.78		
>14 days	16 (7.27)	75.19 ± 7.83		
Combined with other disease types			68.730^2)^	<0.000
0	128 (58.18)	76.82 ± 6.13		
1	66 (30.00)	73.00 ± 7.02		
≥2	26 (11.82)	58.96 ± 10.88		
Stroke frequency			42.225^2)^	<0.000
First time	43 (19.54)	76.56 ± 5.17		
Second time	125 (56.82)	76.10 ± 6.44		
Third time and above	52 (23.64)	65.00 ± 11.36		
Family history			3.195^1)^	0.002
No	164 (74.55)	74.68 ± 8.71		
Yes	56 (25.45)	70.30 ± 9.22		
Type of obstacle			0.073^2)^	0.974
None	76 (34.55)	73.24 ± 9.99		
Limb disorder	71 (32.27)	73.76 ± 7.45		
Visual impairment	38 (17.27)	73.45 ± 10.17		
Dysphagia	35 (15.91)	74.00 ± 8.80		
mRS			21.088^2)^	<0.000
No symptoms	73 (33.18)	77.41 ± 5.71		
Slight disability	80 (36.37)	75.18 ± 6.17		
Moderate disability	19 (8.64)	74.11 ± 7.80		
Moderately severe disability	12 (5.45)	66.75 ± 10.18		
Severe disability to death	36 (16.36)	64.17 ± 12.23		

1) t value, 2) F value; SD: standard deviation. mRS: Categories 0 (No symptoms) and 1 (No significant disability) coalesced into one category: No symptoms; Categories 5 (Severe disability) and 6 (Death) coalesced into one category: Moderate to severe disability.

### 3.4 The relationship between the quality of discharge guidance and discharge readiness for patients with ischaemic stroke

**Figure 5:**
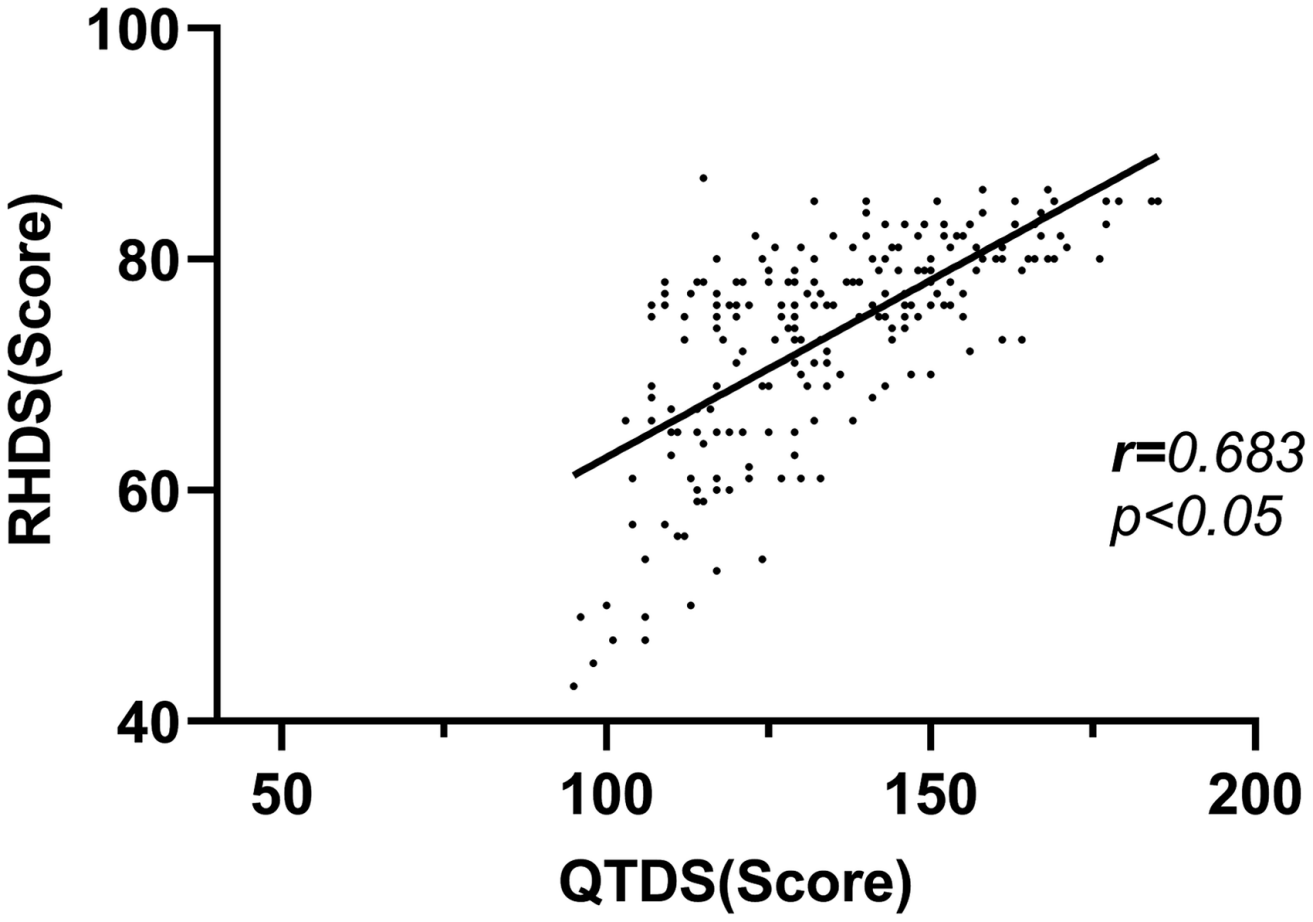
Correlation between discharge readiness and discharge guide quality in patients with ischaemic stroke.

The results showed that the discharge readiness of patients with ischaemic stroke was significantly positively correlated with the total score of discharge guidance quality, the content obtained, the content needed, and the guidance skills and effects (*p* < 0.05). The details are shown in Table 4 and Figure 5.

**Table 4:** Correlation between discharge readiness and discharge guide quality in patients with ischaemic stroke (*r*).

Variable	RHDS	Personal status	Adaptability	Anticipatory support
QDTS	0.683**	0.582**	0.564**	0.565**
Acquired content	0.530**	0.412**	0.465**	0.446**
Required content	0.393**	0.299**	0.398**	0.269**
Guide techniques and effects	0.647**	0.593**	0.479**	0.563**

** *p* < 0.05.

### 3.5 Multiple linear regression analysis of influencing factors of discharge readiness in patients with ischaemic stroke

The total discharge readiness score of patients with ischaemic stroke was used as the dependent variable. Statistically significant general demographic data and variables related to disease-related characteristics (age [years old], income [yuan/month], length of stay, combined with other disease types, stroke frequency, family history, and mRS scores) and QTDS scores were used as independent variables for multiple linear regression analysis. Five variables were entered into the regression equation, which were combined with other disease types, stroke frequency, mRS scores, length of stay, and QTDS scores. R^2^ was 0.680, and the adjusted R^2^ was 0.668, which could explain 66.8% of discharge readiness in patients with ischaemic stroke, as detailed in Table 5.

**Table 5:** Multiple linear regression analysis of influencing factors of discharge readiness in patients with ischaemic stroke.

Variable	Estimate *β*	SE	Standardised *β*	*t*	*p*
(Constant)	49.553	3.090		16.037	<0.000
QTDS	0.212	0.020	0.471	10.773	<0.000
Length of stay	1.274	0.593	0.087	2.148	0.033
Combined with other disease types	−2.976	0.609	−0.230	−4.889	<0.000
Stroke frequency	−2.367	0.591	−0.172	−4.003	<0.000
mRS	−1.407	0.284	−0.220	−4.958	0.001

*F* = 59.422, *P* < 0.05, *R*^2^ = 0.680, adjusted *R*^2^ = 0.668. SE, standard error.

## 4 Discussion

### 4.1 The discharge readiness of patients with ischaemic stroke was at a low level

The present study identified that the overall discharge readiness score for ischaemic stroke patients was (73.56 ± 9.03), with an individual entry score of (6.13 ± 0.75). This indicates a state of inadequate discharge readiness, which is lower than the findings of Zhou (78.17 ± 8.16) (22), which may reflect a concerning trend of reduced discharge preparedness. From a clinical perspective, even modest improvements in readiness scores could translate into meaningful enhancements in patient outcomes, such as reduced confidence in managing rehabilitation at home or in the community, diminished self-care capabilities after discharge, and limited access to post-discharge healthcare information. This is likely associated with the clinical complexity inherent to ischaemic stroke patients and further implies that this population faces considerable challenges in achieving adequate discharge readiness.

This study assessed discharge readiness by measuring three aspects: patients’ personal status, adaptability, and anticipatory support. The lowest individual score (5.37 ± 0.83) pertained to patients’ ability to perform self-care after discharge. This may be attributed to the fact that a majority (54.09%) of study participants were unemployed or laid off, faced a high recurrence rate of the disease, experienced a prolonged recovery period, and had a poor prognosis. These factors coupled with the fear of being unable to manage the disease and rehabilitation treatment, as well as the impact on their work and life, contributed to reduced discharge readiness (23). In response, personalised support development can enhance resilience by improving the ability of ischaemic stroke patients to navigate the health and social care system and stimulating self-management (24). Additionally, incorporating the patient’s perspective with the nurse’s perspective, collaborating with healthcare professionals, providing accessible rehabilitation and post-discharge follow-up support, and ensuring available community and social support can facilitate a smooth transition from hospital to home for stroke survivors and caregivers (25, 26).

### 4.2 There was a correlation between discharge readiness and discharge guidance quality in patients with ischaemic stroke

In this study, the regression coefficient for the quality of the discharge instructions was 0.212 (*p* < 0.001). The readiness of stroke patients for discharge was positively correlated with the quality of discharge instructions, a finding consistent with that of Li et al. (27). This finding may hold potential clinical relevance, as it highlights a modifiable factor—the quality of discharge instructions—which could be further enhanced through targeted interventions such as standardised protocols, staff training, and improved patient education materials (28). In contrast to non-modifiable patient characteristics, such as age, this area may represent a practical opportunity for healthcare services to support better patient outcomes.

Discharge instructions equip patients and their families with the necessary knowledge and skills for disease management and post-rehabilitation care, thereby facilitating a successful transition from hospital to home (29). A randomised controlled trial of a rehabilitation education program, led by the care team and involving shared decision-making among healthcare professionals, patients, and caregivers, demonstrated that patients achieved better motor function recovery post-discharge than those who received traditional rehabilitation (30). This indicates that the expertise, experience, health education competence, and quality of discharge instructions provided by healthcare professionals significantly influence patients’ readiness for discharge. High-quality discharge instructions not only enhance patients’ self-care abilities but also mitigate discrepancies between patient and nurse assessments of discharge readiness. Consequently, the quality of discharge instructions should be prioritised when providing guidance to patients and their families. Effective methods should be employed to deliver relevant knowledge and skills tailored to a patient’s specific circumstances, and patient feedback should be valued to establish a two-way communication model. During hospitalisation, patients and their families should be encouraged to engage in daily disease care and enhance their understanding of the disease and mastery of rehabilitation skills in the later stages, thereby laying a solid foundation for discharge readiness.

### 4.3 The longer the hospital stay, the better the discharge readiness of ischaemic stroke patients

This study demonstrates that ischaemic stroke patients with a hospital stay exceeding 14 days exhibit a significantly higher discharge readiness score (75.19 ± 7.83) compared with those with a hospital stay of less than 7 days (71.60 ± 9.18) and those with a stay of 7–14 days (75.10 ± 8.78). This finding suggests that an extended hospital stay is correlated with enhanced discharge readiness. The underlying reason for this may be that patients with prolonged hospitalisation experience a greater degree of physical function recovery. Conversely, shorter hospital stays may restrict medical staff from providing comprehensive education and instruction to patients and their caregivers, resulting in insufficient discharge preparation. However, a randomised controlled trial has indicated that extended hospitalisation is associated with increased complication rates, higher costs, and diminished functional status at discharge, such as those with long-term medical devices (31, 32). In our study cohort, patients with ischaemic stroke received rehabilitation training and guidance from healthcare professionals following the acute phase. While an extended length of stay may be associated with insufficient rehabilitation resources, the rehabilitation therapy provided likely contributed to improved functional recovery. Furthermore, the mastery of rehabilitation skills appeared to enhance patients’ discharge readiness scores (33–35).

However, in patients requiring rehabilitation, premature discharge may lead to adverse outcomes or readmission, whereas delayed discharge may increase the risk of hospital-acquired adverse events (36). Furthermore, a prolonged hospital stay imposes substantial economic and resource burdens (37). Therefore, healthcare providers should thoroughly evaluate patients’ physical recovery, clinical status, adaptability, and attitudes toward their illness to determine the optimal timing for discharge. This approach ensures patient safety, facilitates adaptation to post-stroke conditions, and promotes successful reintegration into society.

### 4.4 Patients with ischaemic stroke with more types of other diseases, higher stroke frequency, and higher mRs score had lower hospital readiness

This study revealed that ischaemic stroke patients with a higher number of comorbidities exhibited diminished readiness for discharge, underscoring the complexity of recovery in such cases. The long-term prognosis of ischaemic stroke is significantly affected by both comorbidities and disease progression. The regression coefficient for comorbid conditions was −2.976 (*p* < 0.05), indicating a substantial negative impact on discharge readiness. Patients with acute ischaemic stroke often bear a higher burden of comorbidities (38, 39). When patients had more than two comorbid conditions, their readiness for discharge score (58.96 ± 10.88) was markedly lower than that of those with no (76.82 ± 6.13) or one (73.00 ± 7.02) comorbid condition. This may be attributed to the increased severity of the condition in patients with multiple diseases. Additionally, the prolonged course of chronic diseases, such as hypertension and diabetes, necessitates sustained medication adherence and medical treatment, posing both physical and psychological challenges for patients and leading to varying degrees of psychological distress. The increased risk of frailty due to the deterioration of physical function and strength from multiple coexisting diseases contributes to patients being less prepared for discharge. The presence of multiple diseases complicates disease management. Healthcare professionals should provide more personalised guidance and resource support to these patients.

The negative coefficients for stroke frequency (*β* = −2.367, *p* < 0.05) and mRS score (*β* = −1.407, *p* < 0.05) highlight the cumulative impact of functional dependence. Patients who experience recurrent strokes tend to face increased functional impairments, indicating that risk factors such as hypertension and diabetes mellitus may be inadequately managed. These findings emphasise the need for improved secondary prevention strategies and comprehensive long-term disease management.

The mRS is a widely used tool for objectively assessing the severity of ischaemic stroke. It evaluates the degree of dependence and disability in daily activities following a stroke, with higher scores indicating more severe conditions. Patients with ischaemic stroke often experience varying degrees of dysfunction after an event, with greater severity leading to more significant restrictions in normal activities and interpersonal communication. Consequently, these patients require additional resources for rehabilitation support and home care.

## 5 Conclusion

Readiness for discharge among ischaemic stroke patients is inadequate and positively correlated with the quality of discharge guidance. Patients with shorter hospital stays, a higher number of comorbid conditions, increased frequency of stroke occurrence, and higher mRS scores exhibited lower readiness for discharge. These findings suggest that healthcare professionals should enhance the quality of discharge education and provide targeted interventions for patients with shorter hospitalisation and more severe conditions. Additionally, establishing a secondary prevention support mechanism is essential to improve discharge readiness and ensure better post-discharge outcomes for ischaemic stroke patients. This ensures their safe transition from hospital to home.

## 6 Research limitations

One limitation of the study is that the participant population was concentrated in KaiFeng; therefore, the generalisation of the findings may be limited. In the future, observational or experimental studies should be conducted using large sample sizes and multiple centres.

## Data Availability

The raw data supporting the conclusions of this article will be made available by the authors, without undue reservation.
