# Supplementary material for: Level of discharge readiness and influencing factors in ischaemic stroke patients: a descriptive, cross-sectional study
Source: Front Neurol. 2025 Oct 31;16:1683780. doi: 10.3389/fneur.2025.1683780 (PMC12616740; doi:10.3389/fneur.2025.1683780)
Supplement: Supplementary file 1 [file Data_Sheet_1.doc]

**Appendix A. General sociological and disease characteristics**

Dear ischaemic stroke patients, please select the following options based on your actual circumstances.

| **General sociological characteristics** |
| --- |
| 1. Gender: ①Male ②Female |
| 2.Age（years old）: ①≤60 ②＞60 |
| 3.Height：___cm Weight：____kg |
| 4.Education level: ①Junior high school and below ②High school or technical secondary school ③College and above |
| 5.Occupation: ①Work ②Retire ③No job or laid-off |
| 6.Income (yuan/month): ①＜3000 ②3000-5000 ③＞5000 |
| 7. Marital status: ①Unmarried ②Married ③Divorced ④Widowed |
| 8.Medical insurance: ①Resident medical insurance ②Employee medical insurance ③Other medical insurance |
| **Disease characteristics** |
| 9.Length of stay:① < 7 days ②7 days -14 days ③ > 14 days |
| 10.Combined with other disease types：① 0② 1 ③ ≥2 |
| 11.Stroke frequency：①First time②second time③ third time and above |
| 12.Family history：①No②Yes |
| 13.Type of obstacle：①None② Limb disorder ③Visual impairment ④Dysphagia |
| 14.mRS:  ①No symptoms 0  ②No significant disability 1  ③Slight disability 2  ④Moderately disabled  ⑤Moderate to severe disability  ⑥Severe disability to death |

**Appendix B. Readiness for Hospital Discharge Scale (RHDS)**

Dear ischaemic stroke patients, please select the following options based on your actual circumstances.

1. How would you describe your physical pain or discomfort today?

(No pain at all) 0-1 -- 2 -- 3 -- 4 -- 5 -- 6 -- 7 -- 8 -- 9 -- 10 (Severe pain/Severe discomfort)

2. How would you describe your physical strength today?

(Weak) 0 -- 1 -- 2 -- 3 -- 4 -- 5 -- 6 -- 7 -- 8 -- 9 -- 10(Strong)

3. How would you describe your body's energy today?

(Very low vitality) 0 -- 1 -- 2 -- 3 -- 4 -- 5 -- 6 -- 7 -- 8 -- 9 -- 10 (Strong vitality)

4.How would you describe your ability to manage physical self-care upon discharge today?(e.g. hygiene, mobility, toileting)

(Unable to do) 0 -- 1 -- 2 -- 3 -- 4 -- 5 -- 6 -- 7 -- 8 -- 9 -- 10(Completely able to do)

5. How much do you know about the need for self-care when you return home from the hospital?

(Completely unaware) 0 -- 1 -- 2 -- 3 -- 4 -- 5 -- 6 -- 7 -- 8 -- 9 -- 10 (Completely aware)

6. To what extent are you able to handle the demands of life at home?

(Completely unable) 0 -- 1 -- 2 -- 3 -- 4 -- 5 -- 6 -- 7 -- 8 -- 9 -- 10 (Can handle well)

7. To what extent are you able to take personal care of yourself at home? (e.g. hygiene, bathing, toileting, eating, etc.)

(Completely unable) 0 -- 1 -- 2 -- 3 -- 4 -- 5 -- 6 -- 7 -- 8 -- 9 -- 10(Works well)

8. To what extent are you able to administer medical care at home? (e.g. surgical wounds, respiratory therapy, exercise, rehabilitation, taking the right amount of necessary medication at the right time)

(Completely unable) 0 -- 1 -- 2 -- 3 -- 4 -- 5 -- 6 -- 7 -- 8 -- 9 -- 10(Works well)

9. How much emotional support will you receive when you return home from the hospital?

(None) 0 -- 1 -- 2 -- 3 -- 4 -- 5 -- 6 -- 7 -- 8 -- 9 -- 10(Many)

10. How much help can you get with personal care when you return home from the hospital?

(None) 0 -- 1 -- 2 -- 3 -- 4 -- 5 -- 6 -- 7 -- 8 -- 9 -- 10(Many)

11. How much help can you get with home activities after you return home from the hospital~~?~~ (e.g. cooking, cleaning, shopping, child care, etc.)

(None) 0 -- 1 -- 2 -- 3 -- 4 -- 5 -- 6 -- 7 -- 8 -- 9 -- 10(many)

12. How much assistance can you get with your medical care needs when you return home from the hospital? (e.g. Therapy, medicine)

(None) 0 -- 1 -- 2 -- 3 -- 4 -- 5 -- 6 -- 7 -- 8 -- 9 -- 10(many)

**Appendix C. Quality of Discharge Teaching Scale (QDTS)**

Dear ischaemic stroke patients, please select the following options based on your actual circumstances.

1a. How much information would you like to receive from the nurse about self-care at home after discharge?

(None at all) 0 -- 1 -- 2 -- 3 -- 4 -- 5 -- 6 -- 7 -- 8 -- 9 -- 10 (A lot)

1b. How much information have you received from the nurse about self-care at home after discharge?

(None at all) 0 -- 1 -- 2 -- 3 -- 4 -- 5 -- 6 -- 7 -- 8 -- 9 -- 10 (A lot)

2a. How much information did you want from the nurse about post-discharge emotional adjustment/management?

(None at all) 0 -- 1 -- 2 -- 3 -- 4 -- 5 -- 6 -- 7 -- 8 -- 9 -- 10（A great deal）

2b. How much information did you receive from the nurse about post-discharge emotional adjustment/management?

(None at all) 0 -- 1 -- 2 -- 3 -- 4 -- 5 -- 6 -- 7 -- 8 -- 9 -- 10 (A lot)

3a. How much information did you want from the nurse about post-discharge medical care? (e.g. surgical incision care, diet, administration)

Medicine, review, etc)

(None at all) 0 -- 1 -- 2 -- 3 -- 4 -- 5 -- 6 -- 7 -- 8 -- 9 -- 10 (A lot)

3b. How much information did you receive from the nurse about post-discharge medical care?

(None at all) 0 -- 1 -- 2 -- 3 -- 4 -- 5 -- 6 -- 7 -- 8 -- 9 -- 10 (A lot)

4a. How much practice did you think you need in terms of treatment before you leave the hospital?

(None at all) 0 -- 1 -- 2 -- 3 -- 4 -- 5 -- 6 -- 7 -- 8 -- 9 -- 10 (A lot)

4b. How much practice did you get on the treatment before discharge?

(None at all) 0 -- 1 -- 2 -- 3 -- 4 -- 5 -- 6 -- 7 -- 8 -- 9 -- 10 (A lot)

5a. How much information did you want from the nurse about when and who to contact after discharge from the hospital?

(None at all) 0 -- 1 -- 2 -- 3 -- 4 -- 5 -- 6 -- 7 -- 8 -- 9 -- 10 (A lot)

5b. How much information did you get from the nurse about when to contact whom in the hospital after discharge?

(None at all) 0 -- 1 -- 2 -- 3 -- 4 -- 5 -- 6 -- 7 -- 8 -- 9 -- 10 (A lot)

6a. How much information will your family or other personnel need about your care after you return home from the hospital?

(None at all) 0 -- 1 -- 2 -- 3 -- 4 -- 5 -- 6 -- 7 -- 8 -- 9 -- 10 (A lot)

6b. How much information has your family or other personnel received about your care after you return home from the hospital?

(None at all) 0 -- 1 -- 2 -- 3 -- 4 -- 5 -- 6 -- 7 -- 8 -- 9 -- 10 (A lot)

7. How much information did the nurse provide for your special concerns and questions?

(None at all) 0 -- 1 -- 2 -- 3 -- 4 -- 5 -- 6 -- 7 -- 8 -- 9 -- 10 (A lot)

1. To what extent did you think the nurse listened carefully to your concerns?

(None at all) 0 -- 1 -- 2 -- 3 -- 4 -- 5 -- 6 -- 7 -- 8 -- 9 -- 10 (A lot)

9. Was the nurse considerate of your feelings and preferences?

(Not at all) 0 -- 1 -- 2 -- 3 -- 4 -- 5 -- 6 -- 7 -- 8 -- 9 -- 10 (Always)

10.Did you like the way the nurses teach you how to care?

(Not at all) 0 -- 1 -- 2 -- 3 -- 4 -- 5 -- 6 -- 7 -- 8 -- 9 -- 10 (Always)

11.Did you understand the nursing information provided by the nurse

(Not at all) 0 -- 1 -- 2 -- 3 -- 4 -- 5 -- 6 -- 7 -- 8 -- 9 -- 10 (Always)

12. Did the nurse confirm that you understood the information and instructions she provided?

(Not at all) 0 -- 1 -- 2 -- 3 -- 4 -- 5 -- 6 -- 7 -- 8 -- 9 -- 10 (Always)

1. Did you receive consistent information from the nursing and medical staff?

(Not at all) 0 -- 1 -- 2 -- 3 -- 4 -- 5 -- 6 -- 7 -- 8 -- 9 -- 10 (always)

14. Did the nurse always provide information to you at the appropriate time

(Not at all) 0 -- 1 -- 2 -- 3 -- 4 -- 5 -- 6 -- 7 -- 8 -- 9 -- 10 (always)

15. Did the nurse consistently provide information at a time when your family was able to participate?

(Not at all) 0 -- 1 -- 2 -- 3 -- 4 -- 5 -- 6 -- 7 -- 8 -- 9 -- 10 (Always)

16. Did the nurse help you build confidence in self-care at home?

(Not at all) 0 -- 1 -- 2 -- 3 -- 4 -- 5 -- 6 -- 7 -- 8 -- 9 -- 10 (Largely)

17. Are you confident that you will be able to deal with any medical emergencies you may encounter after discharge?

(not at all) 0 -- 1 -- 2 -- 3 -- 4 -- 5 -- 6 -- 7 -- 8 -- 9 -- 10 (Largely)

18. Did the information provided by the nurse reduce your anxiety about leaving the hospital for self-care at home?

(not at all) 0 -- 1 -- 2 -- 3 -- 4 -- 5 -- 6 -- 7 -- 8 -- 9 -- 10 (Largely)
